# Comparing the Effects of Whey Extract and Casein Phosphopeptide-Amorphous Calcium Phosphate (CPP-ACP) on Enamel Microhardness

**Published:** 2015-03

**Authors:** Mohammad Bagher Rezvani, Mehrdad Karimi, Raheleh Akhavan Rasoolzade, Roza Haghgoo

**Affiliations:** a Dept. of Reparative Dentistry, Dental School, Shahed University, Tehran, Iran.; b PHD Traditional Medicine, Traditional Medicine School, Tehran University of Medical Sciences, Tehran, Iran.; c Resident, Dept of Periodontics, Dental School, Shahid Beheshti University of Medical Sciences, Tehran, Iran.; d Dept. of Pediatric Dentistry, Dental School, Shahed University, Tehran, Iran.

**Keywords:** Demineralization, Extracts, Microhardness, Remineralization, Whey

## Abstract

**Statement of the Problem:**

With the recent focus of researches on the development of non-invasive treatment modalities, the non-invasive treatment of early carious lesions by remineralization would bring a major advance in the clinical management of these dental defects. Casein phosphopeptide-amorphous calcium phosphate (CPP-ACP) is considered to be effective in tooth remineralization.

**Purpose:**

The aim of this *in-vitro* study was to compare the effects of whey and CPP-ACP in increasing the enamel microhardness.

**Materials and Method:**

Microhardness of 30 sound human permanent premolars was measured before and after 8-minute immersion of samples in Coca-Cola. The teeth were then randomly divided into 3 groups and were immersed in artificial saliva, whey, and tooth mousse for 10 minutes. The changes of microhardness within each group and among the groups were recorded and analyzed using paired t-test.

**Results:**

The microhardness increased in each group and between the groups; this increase was statistically significant (*p*= 0.009). **

**Conclusion:**

The effect of whey on increasing the enamel microhardness was more than that of tooth mousse.

## Introduction

Dental erosion has recently been in the center of attention in many developed countries. [1] Dental erosion is defined as partial demineralization of enamel or dentine resulted from chemical agents without involvement of microorganisms. [2] Clinical features of dental erosion include the loss of tooth, leaving shiny surface and flattening of the convex surfaces. With continued acid exposure, concavities and grooves will appear on the smooth or incisal/occlusal surfaces. [3] The consequences of dental erosion demand its treatment. With the substantial significance of conservative dentistry, many studies have evaluated different materials to aid in remineralization of demineralized parts of the involved tooth. [4-7]

Phosphate-containing calcium compounds are the most similar materials to hydroxyapatite, both chemically and structurally, which have the advantages of biocompatibility, high chemical stability, and wear resistance. [8-9] Casein phosphopeptide-amorphous calcium phosphate (CPP-ACP) has been added to different pharmaceutical products and foods and has been claimed to be effective in tooth Remineralization. [1] The dairy products containing CPP-ACP have been introduced to be effective in preventing dental caries since 1950. [10] Several studies have shown that dairy products such as milk, yogurt and cheese extract are effective in preventing dental caries. [11-12] The findings of a study showed that milk reduced the cariogenic potential of sugar-containing foods. [11] Another study reported that children who were caries-free for a 2-year period had more cheese consumption, compared to those children with more dental caries. [12] The heavy part, the precipitation of yogurt after centrifugation in test tube is considered as whey. Whey as a dairy product which contains CPP-ACP might be used to repair early cavities. The aim of this *in-vitro* study was to compare the effects of whey extract and CPP-ACP on the enamel microhardness.

## Materials and Method

Thirty sound human premolars were selected (sample size was based on the pilot study) and were mounted in a special frame, made of polyester material. Samples were polished until a smooth and uniform surface was achieved. The initial microhardness was then measured and recorded by employing the Vickers microhardness system (Shimadzu; M- g5037, Japan) using a force equivalent to 50 grams on three points. The samples were soaked in 40 ml of Coca-Cola (PH =4.7) for 8 minutes. The microhardness was again measured without washing the samples. The beverage was poured in the sample container immediately after opening the bottle cap. To keep the gas and to reduce the buffering action of the ions which were dissolved from the enamel surface, the beverage was exchanged every 2 minutes (totally 4 times). [9] Then the teeth were mounted and randomly divided into the following groups:

1. Group C (control): the teeth were immersed in artificial saliva (Kin Hidrat spray; Spain) for 10 minutes. [7]
2. Group W (whey): the teeth were immersed in whey (heavy and precipitated portion of yogurt) for 10 minutes. [7]
3. Group TM (tooth mousse): the teeth were immersed in tooth mousse (GCMI paste plus; Japan) for 10 minutes. [7]

The changes in microhardness, within each group and among the groups, were evaluated by using paired t-test and two-way ANOVA test with SPSS Software, Version 14, and adopting a significant level of 0.05. Multiple comparisons were also performed using the Bonferroni method. 

## Results

After 8 minutes of immersion, an average of 34.6% reduction of microhardness was recorded in samples. The microhardness of the samples after immersion in artificial saliva, whey and tooth mousse in the second phase had a statistically significant increase; this increase was statistically significant among the experimental groups (*p*= 0.009). The increase of microhardness was 30% in the whey group, 17% in artificial saliva group, and 8% in the tooth mousse group. Multiple comparisons using the Bonferroni method revealed that the increase of microhardness in the whey group (61.3) was significantly higher than that in the artificial saliva group (42.3) (*p*= 0.010). The difference in the increase of microhardness between the whey group (61.3) and tooth mousse group (22.7) was nearly significant (*p*= 0.074) (Table 1, 2).

**Table 1:** Changes in the rate of microhardness in experimental groups

**Experimental groups**	**Number**	**Primary evaluation**		**After demineralization**		**After intervention**	
		**Standard ** **deviation**	**Mean**	**Standard ** **deviation**	**Mean**	**Standard ** **deviation**	**Mean**
Artificial saliva	30	64.6	370.9	45.1	239.6	41.6	281.8
Tooth mouse	30	86.3	361.5	50.4	243.2	37.3	265.8
Whey	30	93.4	319.1	38.9	204.0	66.3	256.3

**Table 2:** Multiple comparisons based on the Bunfereny method

**Experimental groups**	**Experimental ** **groups**	**Difference of** **mean of changes**	**Error**	**Significance**	**Confidence interval 95%**	
					**Lower limit**	**Higher limit**
Artificial saliva	Tooth mouse	6.2050	8.76577	1.000	-14.9826	27.3926
	Whey	26.0833^*^	8.76577	.010	4.8958	47.2709
Tooth mouse	Artificial saliva	-6.2050	8.76577	1.000	-27.3926	14.9826
	Whey	19.8783	8.76577	.074	-1.3092	41.0659
Whey	Artificial saliva	-26.0833^*^	8.76577	.010	-47.2709	-4.8958
	Tooth mouse	-19.8783	8.76577	.074	-41.0659	1.3092

Based on observed means.

The error term is Mean Square (Error) = 2305.164.

*. The mean difference is significant at the 0.05 level.

## Discussion

CPPs are phosphorylated casein-derived peptides and it has been stated that CPP-ACP can re- mineralized enamel lesions. [13] The remineralization activity of CPPs is because of their ability in stabilizing high levels of ACP on the tooth surface, preventing the demineralization process and consequently, increasing the remineralization of enamel caries. [1 , 13]

In this study, the remineralization potential of CPP-ACP and the heavy and precipitated portion of yogurt on enamel microhardness were evaluated for the first time. Other studies used the floating part whilst the heavy and precipitated portion of the yogurt (as whey) was evaluated in the current study.

Based on the results of the present study, the microhardness of samples decreased after immersion in Coca-Cola. This finding is consistent with the studies of Haghgoo and Foruzeshtabar [14] and Tantbirojn *et al.* [1]

 The findings of this study showed that artificial saliva, tooth mousse and whey increased the microhardness of teeth. The increase in the microhardness was significantly different among the experimental groups. The increase of microhardness was significantly higher in the whey group compared to the other two groups.

This study also revealed that microhardness was increased more in saliva group rather than the tooth mousse group. Artificial saliva is composed of calcium chloride, potassium phosphate and other minerals that might affect the tooth Remineralization. [15]

A study by Tantbirojn *et al.* found similar results; [1] with the exception that they studied the bovine teeth, whereas the current study evaluated human teeth. Tantbirojn *et al.* sectioned the teeth; the increase in the heat and the pressure that was produced because of sectioning the teeth affected the microhardness. [1] Moreover, the saliva substitute was different in previous studies.

Regarding the results obtained by Ferrazzano and Cantile’s study, natural CPP in probiotic yogurt is effective in preventing demineralization of dental enamel and enhancing the remineralization process. [16] In their study, immersion in remineralising solution (probiotic yogurt) resulted in absorption of calcium from the solution. It is worth mentioning that the type of whey used in previous studies was different. Ferrazzano and Cantile evaluated the floating part of the surface of test tube containing yogurt as remineralising solution; while in the present study the heavy and precipitated portion of yogurt was used as whey.

Harper *et al.* stated that calcium, phosphorus, and whey in casein-free milk have favorable effects on reducing dental caries. [17] Their findings are in line with the results of the present study. In this study, the effect of tooth mousse was lower, compared to other studies. The reason for this diversity can be due to the different methodology used. In most previous studies, the effect of tooth mousse has been evaluated in-vitro and in intermittent periods, [16 - 17] while we studied the effect of CPP-ACP in one step and for 10 minutes.

Lijima *et al.* showed that chewing CPP-containing gum increased the normal enamel density. [18] It seems that the short time of evaluation in the present study, compared with previous studies, has affected the results. Lijima’s study evaluated the effect of tooth mousse micro-radiographically and this difference in methodology makes it difficult to compare the results of their studies with the findings of the current one.

The results of this study and previous studies have demonstrated the effective role of CPP-ACP (in the form of tooth mousse) in remineralization of enamel. [16 - 18]

Srinivasan *et al.* compared the remineralization of softened enamel of human teeth after exposure to the pastes containing CPP-ACP and CPP-ACP with 900 ppm fluoride. [19] They showed that CPP-ACP and CPP-ACP with 900 ppm fluoride substantially remineralized the softened enamel and the remineralization potential of CPP-ACP with fluoride was higher than that of CPP-ACP per se. The results yielded by the study of Srinivasan *et al.* are similar to the findings of the present study, however their study was performed in-vitro and the synergic effect of fluoride was investigated. [19]

The study performed by Giulio *et al.* showed that topical applications of CPP-ACP could be effective in enamel Remineralization, [20] which is consistent with the results of our study. Giulio *et al.* evaluated the remineralization potential of CPP-ACP on stripped enamel morphology after being exposed to an acid solution while we studied the same potential on the eroded enamel.

Panich and Poolthong evaluated the effect of CPP-ACP paste on the enamel hardness in an in-vitro study and found results similar to our study. The two studies were different in that they studied re-hardening of the buccal surfaces of incisors, [21] while we compared the effects of whey extract and CPP-ACP on enamel microhardness of premolars.

Cai *et al.* evaluated the effects of CPP-ACP, associated with a fruit-flavored sugar-free chewing gum containing citric acid on the enamel remineralization and the acid resistance of remineralized enamel. [22] The results of that study showed that the remineralization potential of chewing gum containing CPP-ACP is significantly higher than chewing gum without CPP-ACP. [22] This finding is consistent with the results obtained by our study. The design of Cai *et al.’s* study was *in vitro* and microradiographic technique was employed to detect the level of remineralization. In their study, the remineralization potential of CPP-ACP after acid-induced demineralization was evaluated while we studied the remineralization of simulated erosive lesions.

Shen *et al. *determined the ability of CPP-ACP in remineralization of the enamel subsurface lesions in human teeth. [23] The results of the current study is similar to the findings of their  study, although, we studied the effect of CPP-ACP and heavy and precipitated part of yogurt used as whey on remineralization of erosive lesions. Future studies are suggested to compare the heavy and precipitated part with the floating part of yogurt on the enamel re-hardening of the erosive lesions. It is also suggested to evaluate the remineralization ability of the above agent (heavy and precipitated part with the floating part of yogurt) on enamel subsurface lesions.

## Conclusion

Based on the results of this study, enamel microhardness of permanent human premolars was significantly increased following the application of materials that consist of calcium and phosphate such as whey and CPP-ACP. The effect of whey on enamel microhardness was more than CPP-ACP (tooth mousse). 
